# Circadian rhythms and psychiatric profiles in young adults with unipolar depressive disorders

**DOI:** 10.1038/s41398-018-0255-y

**Published:** 2018-10-09

**Authors:** Rébecca Robillard, Joanne S. Carpenter, Naomi L. Rogers, Sarah Fares, Ashlee B. Grierson, Daniel F. Hermens, Sharon L. Naismith, Sharon J. Mullin, Kristy-Lee Feilds, Nick Glozier, Elizabeth M. Scott, Ian B. Hickie

**Affiliations:** 1 The Royal’s Institute of Mental Health Research, 1145 Carling Ave, Ottawa, ON K1Z 7K4 Canada; 2 School of Psychology, University of Ottawa, Ottawa, ON K1N 6N5 Canada; 3 Concord Clinical School, The University of Sydney, Gate 3 - Hospital Road, Concord Repatriation General Hospital, Sydney, NSW 2139 Australia; 4 Clinical Research Unit, Brain & Mind Centre, The University of Sydney, 94 Mallett St, Camperdown, NSW 2050 Australia; 5 Healthy Brain Ageing Program, Faculty of Science, Charles Perkins Centre and Brain & Mind Centre, The University of Sydney, Sydney, NSW Australia

## Abstract

Abnormalities in circadian rhythms have been reported in people with mood disorders, but these abnormalities are marked by considerable inter-individual variability. This study aimed to identify pathophysiological subgroups on the basis of circadian markers and evaluate how these subgroups relate to psychiatric profiles. Thirty-five young adults (18–31 years old) receiving clinical care for unipolar depressive disorders and 15 healthy controls took part to this study. The Hamilton Rating Scale for Depression and the Young Mania rating scale were used to evaluate the severity of mood symptoms in participants with depressive disorders. All participant underwent ambulatory sleep monitoring with actigraphy for about 12 days before attending a laboratory-based chronobiological assessment which included repeated salivary samples to determine dim light melatonin onset (DLMO) and continuous core body temperature (CBT) monitoring using an ingestible temperature sensor. Cluster analyses were conducted across all participants to identify subgroups with consistent circadian timing profiles based on DLMO and the nocturnal minima of CBT. Two clusters were identified: ‘delayed’ and ‘conventional timing’ circadian phase. Descriptive analyses showed that the delayed cluster was characterised by abnormal time relationships between circadian phase markers and the sleep–wake cycle. Importantly, individuals from the delayed cluster had worse depression severity (*t*(28) = −2.7, *p* = 0.011) and hypomanic symptoms (*Z* = −2.2, *p* = 0.041) than their peers with conventional circadian timing. These findings suggest that delayed and disorganised circadian rhythms may be linked to worse psychiatric profiles in young people with depressive disorders.

## Introduction

The biological clock in the suprachiasmatic nuclei regulates the temporal coordination of physiological rhythms to optimise brain and body functions across the different phases of the 24-h cycle. The temporal organisation of these rhythms is important for several aspects of physical and mental health (for a review, see ref.^1^). For instance, experimentally induced alterations in the temporal relationship between the light–dark cycle and circadian rhythms can induce mood impairments^2,3^. It has thus been hypothesised that circadian rhythms disruption may play a role in the pathophysiology of mood disorders (e.g. refs. ^4,5^).

In individuals with normally entrained circadian rhythms, dim light melatonin onset (DLMO) occurs about 2–3 h prior to bedtime and core body temperature (CBT) reaches a nadir about 1–3 h before wake-up time^6,7^. The internal synchrony between endogenous rhythms underpins robust and coherent circadian rhythmicity. In addition, the temporal alignment of endogenous rhythms with the 24-h patterns of sleep, wakefulness, feeding and physical activity supports variations in physiological functions at times that best match behavioural schedules. This intricate temporal organisation can be characterised by the time intervals between circadian phase markers (i.e. phase angles).

The nature of circadian disturbances in affective disorders still remains unclear (for a recent review, see ref. ^8^). Some studies reported phase advances in the rhythm of melatonin and CBT^9,10^, whereas others reported phase delays^11–14^. One of the few studies assessing circadian organisation in people with depression reported prolonged phase angles between DLMO and CBT nadir^15^. Additionally, depression severity and later stages of affective disorders have been reported to correlate with shorter phase angles between DLMO and sleep timing^16,17^ and with longer phase angles between sleep midpoint and CBT nadir^15^. Previous findings, however, suggest considerable inter-individual variability in circadian rhythms across people with depressive disorders^15,16,18^, which may result from the co-existence of subgroups with distinct circadian profiles. For instance, a previous study in seasonal depression identified a subgroup of individuals with a short phase angle between melatonin onset and the sleep–wake cycle, who had stronger associations between circadian misalignment and depressive symptoms than individuals with longer phase angles^19^. To better understand the heterogeneity of circadian disturbances in the context of depression, there is a need to examine profiles of circadian organisation beyond global group comparisons and to determine how these profiles may relate to psychopathological symptomatology.

The clinical relevance of subthreshold hypomanic symptoms emerging the context of unipolar depression is increasingly recognised^20,21^. A previous study based on a sample of people with anxiety, depressive and bipolar disorders suggested that, independently of diagnosis, lower circadian amplitude and rhythmicity of the sleep–wake cycle correlated with worse manic/hypomanic symptoms^22^. Similar findings have been reported in a mixed sample of people with depression and people without psychiatric disorders; with later and more irregular rest–activity rhythms being linked to worse manic/hypomanic and depressive symptoms. Whether the severity of both depressive and manic symptoms relate to the circadian rhythms of melatonin and CBT remains to be further investigated in people with unipolar depression.

The present study sought to identify circadian profiles linked to depression and evaluate how they relate to mood dysfunctions in young adults. Firstly, data-driven methods were applied in a sample of young adults with depressive disorders intermixed with healthy controls in order to identify consistent subgroups based on circadian phase. Secondly, phase angles were compared across these circadian subgroups to characterise patterns of circadian organisation for descriptive purposes. Finally, these circadian subgroups were compared in terms of the proportions of individuals with depressive disorders and the severity of mood symptoms. Considering the normal age-related phase delay occurring during adolescence and often persisting in young adulthood, we anticipated that a subgroup of participants would have delayed circadian rhythms, especially those with depressive disorders. We further hypothesized that this circadian delay would be linked to abnormal circadian phase angles and worse psychiatric symptoms.

## Materials and methods

### Participants

This study included 50 participants between 18 and 31 years of age: 35 persons receiving care for unipolar depressive disorders recruited from early intervention youth services at the clinics of the Brain and Mind Centre, Australia, and 15 healthy controls recruited from the community. Sample characteristics are reported in Table 1. On average, the control group was 3.2 years older than the depressive disorder group, but there was no significant difference in sex distributions.

**Table 1:** Diagnostic groups characteristics

	Control	Depression	*U*/*χ*^2^/*t*	*p*
*n*	15	35		
Age, mean years (SD)	24.3 (3.4)	21.1 (2.9)	*U* = 122.0	0.003
Sex, % females	8 (53.3%)	21 (60.0%)	*χ*2 = 0.19	0.662
*Circadian phase marker*				
DLMO	22.06 (1.49)	23.58 (2.45)	*U* = 135.0	0.007
CBT_min_	4.19 (1.32)	5.17 (2.15)	*t* = 1.5	0.139
*Sleep*				
Sleep_ON_	0.13 (0.55)	1.14 (1.59)	*t* = 2.4	0.021
Sleep_OFF_	7.54 (1.05)	9.49 (2.01)	*t* = 4.2	0.000
TST (min)	431.6 (53.5)	445.1 (56.2)	*t* = 0.7	0.483
Sleep efficiency (%)	90.8 (2.5)	85.1 (4.0)	*t* = −4.6	0.000
*Phase angles, mean (SD); min*				
Sleep_ON_−DLMO	2.1 (1.4)	1.2 (2.2)	*t* = −1.4	0.169
CBT_min_−Sleep_mid_	0.6 (1.2)	−0.2 (1.7)	*t* = −1.6	0.107
CBT_min_−DLMO	6.2 (1.2)	5.2 (2.5)	*t* = −1.9	0.060
*Occupational status*, *n* (%)				
FT employment	3 (21.4%)	4 (13.3%)	*χ*2 = 0.47	0.494
Studying and PT employment	5 (35.7%)	7 (23.3%)	*χ*2 = 0.74	0.390
Studying	5 (35.7%)	7 (23.3%)	*χ*2 = 0.74	0.390
PT Employment	1 (7.1%)	7 (23.3%)	*χ*2 = 1.7	0.195
Volunteering	0 (0%)	1 (3.3%)	χ2 = 0.48	0.490
Unemployed	0 (0%)	4 (13.3%)	*χ*2 = 2.1	0.152
*Psychotropic medication*, *n* (%)				
None	–	16 (45.7%)		
Antidepressants	–	15 (42.9%)		
Mood stabilisers	–	1 (2.9%)		
Antipsychotics	–	5 (14.3%)		
Stimulants	–	1 (2.9%)		

Occupational data was missing for 5/35 depression cases and 1/15 controls

*SD* standard deviation, *FT* full time, *PT* part time

All participants with depressive disorders were assessed by a mental health professional using DSM-IV criteria to determine all psychiatric diagnoses. To be included in the study these participants had to hold a diagnosis of major depressive disorder (*n* = 18) or depressive disorder not otherwise specified (*n* = 17). Twelve participants had one or more of the following psychiatric comorbidities: dysthymia (*n* = 1), adjustment disorder (*n* = 1), anxiety disorder (*n* = 4), personality disorder (*n* = 3), eating disorder (*n* = 1), substance abuse (*n* = 1), and somatisation disorder (*n* = 1). In the depression group, 54.3% of participants were taking psychotropic medications (Table 1). Participants taking hypnotics, benzodiazepines, or melatonin-based medication were systematically excluded. None of the participants reported any respiratory or neurological disorders, or had done regular shift-work or transmeridian travel within 60 days prior to study entry. None of the control participants reported any current mental disorder and none were taking psychotropic medications. Neither comorbidities, nor psychotropic medications significantly influenced the circadian or mood variables studied herein (see supplemental table).

All participants gave written informed consent prior to starting the study. The study protocol was approved by the Human Research Ethics Committee of the University of Sydney. Eighteen participants from the depressive disorder group were also included in a previous brief report on sleep–wake and melatonin rhythms in mood disorders^12^.

### Procedures

#### Clinical assessment

For participants with depressive disorders, standardized structured clinical interviews were conducted by a research psychologist. The severity of depressive symptoms was rated on the 17 items of the Hamilton Rating Scale for Depression (HRSD)^23^. This allowed the quantification of symptoms severity along four factors: anxiety, core depressive symptoms, sleep disturbances, and somatisations. Total HRSD score was calculated without the sleep items to ensure that potential group differences on HRSD were not solely driven by differences in sleep. The Young Mania Rating Scale (YMRS) was also administered to assess subthreshold hypomania symptoms. HDRS data was missing for five participants, and YMRS data was missing for 11 participants.

#### Sleep–wake cycle monitoring

Sleeping patterns were estimated with actigraphy monitoring over an average of 12.9 ± 2.7 days (Actiwatch-64/L/2, Philips Respironics, USA or GENEActiv, Activinsights, UK). All data were visually inspected by trained technicians to adjust the start and end of each sleep episode.

Sleep onset (Sleep_ON_) and the midpoint of the sleep episode (Sleep_mid_) were averaged over the monitoring period and used to calculate phase angles. Mean sleep offset (Sleep_OFF_), total sleep time and sleep efficiency (i.e. total sleep time divided by time in bed) were also computed for descriptive purposes.

#### Circadian rhythms assessment

Following the period of actigraphy monitoring, participants were brought into the laboratory for an overnight assessment starting 8 h prior to their habitual sleep-onset time. Circadian rhythms were measured using a protocol in which the timing of all measurements was based on each individual’s mean actigraphic sleep schedule. Participants were invited to go to sleep 2.5 h after habitual bedtime, were woken up at their habitual wake time, and remained in the laboratory in the morning for the following 3.5 h. Food or beverages containing caffeine were prohibited from noon onward on the circadian assessment day. During their stay in the laboratory, participants were kept under dim light (<30 lx), remained seated with their feet elevated on a footstool (except when they had to go to the toilet), and were fed temperature-controlled snacks in the evening (three snacks 2.5–3.25 h apart) and morning (two snacks about 1 h apart).

#### Salivary melatonin

In line with standard partial melatonin sampling protocols (e.g. refs. ^24,25^), saliva samples were collected with Salivette tubes (Sarstedt, Nümbrecht, Germany) every 30 min over an 8.5-h period in the afternoon and evening (i.e. until 2 h after habitual sleep onset). Salivary melatonin (200 μl) was assayed in duplicate by double antibody radioimmunoassay (Buhlmann Laboratories AG, Schönenbuch, Switzerland)^26^ with a detection threshold of 0.999 pg/mL (inter-assay coefficient of variation between 8.2% and 15%, intra-assay coefficient: <10.0% across the standard curve). DLMO was defined by interpolation (e.g. ref. ^27^) based on the two samples surrounding the sample where salivary melatonin concentration reached a threshold of 3 pg/mL and remained above this threshold for the three subsequent samples. If melatonin levels did not reach this threshold before the end of the saliva sampling period, DLMO was estimated to occur 30 min after the timing of the last sample, that is 2.5 h after habitual sleep onset (this was the case for five participants: one from the control group and four from the depression group).

#### Core body temperature

CBT was measured across the evening, night, and morning with an ingestible capsule-size sensor and the EquivitalTM LifeMonitor (Equivital, Cambridge, UK). For six participants, CBT data was recorded using the same ingestible sensor and the VitalSense monitor (Philips Respironics, Bend, USA) while participants slept at their habitual sleep schedule on a separate laboratory visit (i.e. 1 week after salivary melatonin sampling).

For all participants, CBT data points for each 60 s were used to generate a partial circadian curve. The first hour of recording was excluded to avoid the initial variations related to gastrointestinal transit. To reject artifacts, all data points where CBT rose or dropped more than 0.15 °C from the previous minute were systematically excluded. The following 30 min during which temperature moved back towards its normal level were also excluded and further visual inspection was conducted to exclude remaining artefacts. On average, 10% of individual CBT data was missing due to temporary signal loss or artifacts rejection, and CBT datasets retained 15.5 h of valid recording. CBT nadir (CBT_min_) was determined using Prism software (GraphPad Software, La Jolla, USA) as the timing of the minimum of the best-fit curve based on a sine wave function with an absolute frequency <0.5 Hz constraint.

### Statistical analyses

For all analyses, outlying data points were adjusted by curtailing (±2 SD from the mean; this affected 1 (2%) to four participants (8%) for sleep and circadian variables, and two participants (8%) for the YMRS). Normality of distributions were assessed with Shapiro–Wilk tests and non-parametric tests were used for variables which were not normally distributed. For all parametric tests, when the Levene’s test suggested that variances across groups were not homogeneous, calculations were done using un-pooled variances and a corrected degrees of freedom. Statistical analyses were conducted with the Statistical Package for Social Sciences (IBM SPSS Statistics for Windows, Version 20.0. Armonk, USA) and the R package (version 3.2.3. Vienna, Austria).

Prior to inclusion in cluster analysis, data were converted to *z*-scores. *K*-means cluster analysis based on DLMO and CBT_min_ was conducted across all participants. The Hartigan method was used to determine the optimal number of clusters^28^.

For descriptive purposes, age, sex distribution, and the proportion of participants with and without depressive disorders were compared across the clusters with a two-tailed Mann–Whitney *U* test and two-sided Chi-square tests. Also, to characterise the nature of the resulting clusters, independent sample two-tailed *t*-tests were used to compare parameters of the sleep–wake cycle, circadian phase markers and the following circadian phase angles: Sleep_ON_−DLMO, CBT_min_−Sleep_mid_, and CBT_min_−DLMO.

The central analyses evaluated differences in HDRS and YMRS across clusters using independent sample two-tailed *t*-tests or two-tailed Mann–Whitney *U* test.

## Results

### Descriptive cluster characteristics

A two-cluster solution was found (see Table 2 for cluster characteristics and statistics). Based on the variables included in the cluster analysis, one of the clusters was characterised by later DLMO and CBT_min_ (i.e. ‘delayed’ cluster) compared to the other cluster (i.e. ‘conventional timing’ cluster). More precisely, DLMO and CBT_min_, respectively, occurred 3.7 and 3.0 h later in the ‘delayed’ cluster compared to the ‘conventional timing’ cluster.

**Table 2:** Cluster groups characteristics

	Conventional cluster	Delayed cluster	*U*/*t*/*χ*2	*p*
*n*	33	17		
Age, mean (SD)	21.9 (3.4)	22.4 (3.4)	*U* = 252.5	0.564
Sex, females *n* (%)	21 (64%)	8 (47%)	*χ*2 = 1.3	0.261
Diagnostic group, depression/control	21/12	14/3	*χ*2 = 1.9	0.171
*Circadian phase marker*				
DLMO	22.10 (1.30)	1.49 (2.44)	*t* = 5.1	<0.001
CBT_min_	3.58 (1.25)	6.59 (1.45)	*t* = 6.5	<0.001
*Sleep*				
Sleep_ON_	24.09 (1.12)	2.28 (1.47)	*t* = 5.3	<0.001
Sleep_OFF_	8.33 (1.28)	10.35 (2.12)	*t* = 3.3	0.003
TST (min)	455.9 (45.6)	411.5 (60.6)	*t* = −2.5	0.017
Sleep efficiency (%)	88.0 (4.4)	85.5 (4.3)	*t* = −1.6	0.112
*Phase angles, mean (SD) (min)*				
Sleep_ON_−DLMO	118.3 (78.8)	31.2 (167.0)	*t* = −2.0	0.063
CBT_min_-Sleep_mid_	−17.8 (83.7)	46.4 (106.5)	*t* = 2.3	0.028
CBT_min_-DLMO	347.3 (111.1)	299.7 (168.2)	*t* = −1.1	0.303

*SD* standard deviation, *DLMO* dim light melatonin onset, *CBT*_*min*_ core body temperature mimimum, *Sleep*_*ON/OFF*_ sleep onset/offset, *TST* total sleep time, *Sleep*_*mid*_ midpoint of the sleep period

There was no significant age or sex distribution difference across clusters (Table 1). Fourteen (40%) of the 35 participants with depressive disorders and 3 (20%) of the 15 healthy controls were classified in the ‘delayed’ cluster, but these proportions did not differ significantly (*χ*^2^ = 1.9, *p* = 0.171). Of those with depressive disorders and other psychiatric comorbidities, 50% (*n* = 6) were classified in the ‘delayed’ cluster and 50% (*n* = 6) in the ‘conventional timing’ cluster (*χ*^2^ = 1.8, *p* = 0.180). There was a slight, but non-significant difference in cluster classification based on psychotropic medication intake: 68% (*n* = 13) of individuals taking psychotropic medications were classified in the ‘delayed’ cluster and 32% (*n* = 6) in the ‘conventional timing’ cluster (*χ*^2^ = 1.2, *p* = 0.268).

In addition to marked delays in DLMO and CBT_min_, the ‘delayed’ cluster had significant, yet milder, delays in the timing of sleep onset and sleep offset, and shorter total sleep time when compared to the ‘conventional timing’ cluster. The ‘delayed’ cluster also had a significantly longer phase angle between CBT_min_ and sleep_mid_, and tended to have a shorter phase angle between DLMO and sleep onset time than the ‘conventional timing’ cluster. Inspection of individual data showed that DLMO occurred after rather than before habitual sleep time (i.e. inverted phase angle) in 44% (*n* = 7) of people classified in the ‘delayed’ cluster with valid phase angle data.

Eighty-five percent (*n* = 6) of these individuals from the ‘delayed’ cluster with inverted phase angles were from the depression group.

### Psychiatric symptom severity and circadian profile

**Fig. 1:**
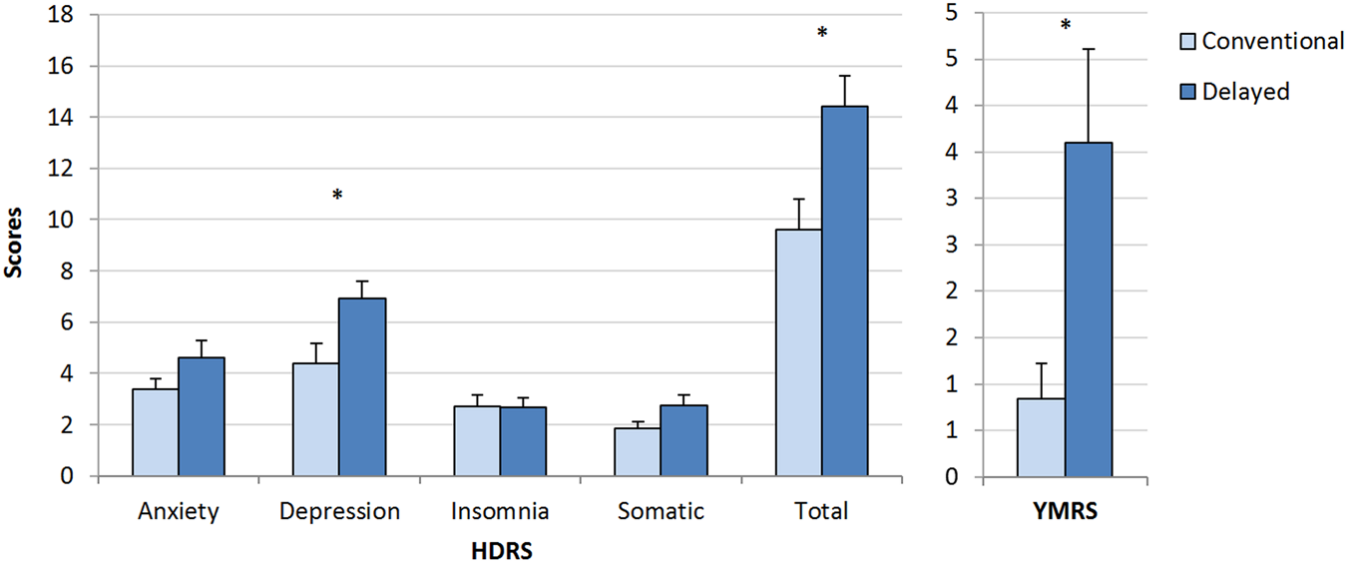
Clinical profile of individuals with depressive syndromes across cluster groups. Means and standard errors of the mean for: (**a**) Left panel—Hamilton Depression Rating Scale (HDRS) subscales and total score (minus the sleep items); conventional cluster: *n* = 18, delayed cluster: *n* = 12 and (**b**) right panel—the Young Mania Rating Scale (YMRS); conventional cluster: *n* = 13, delayed cluster: *n* = 11. *p < 0.050

Figure 1 shows mean scores on the HDRS and YMRS in participants with depressive disorders from each cluster. Compared to the ‘conventional timing’ cluster, the ‘delayed’ cluster had a significantly higher HDRS total score (minus the sleep items; *t*(28) = −2.7, *p* = 0.011). Scores on the depression subscale were also significantly higher in the ‘delayed’ than in the ‘conventional timing’ cluster (*t*(28) = 2.3, *p* = 0.031). No significant cluster difference or trends were found for the anxiety, insomnia, or somatic HDRS subscales. Compared to the ‘conventional timing’ cluster the ‘delayed’ cluster had significantly higher YMRS total scores (*Z* = −2.2, *p* = 0.041).

## Discussion

This study used data-driven methods to identify consistent subgroups of young people with unipolar depressive disorders based on circadian phase markers. A profile suggestive of delayed circadian rhythms was found in 40% of individuals from the depressive disorders group. This subgroup with delayed rhythms also presented significant abnormalities in circadian organisation, as well as worse mood symptoms than individuals with conventional circadian timing.

In the ‘delayed’ cluster, the magnitude of the delays in evening melatonin secretion and nocturnal CBT_min_ was greater than that of the delay in sleep schedules, translating into some degree of temporal misalignment between the circadian rhythms of melatonin and CBT, and the sleep–wake cycle. In fact, in a subgroup of those with affective disorders, the normal time relationship between evening melatonin release and habitual sleep time was completely inverted, with DLMO occurring after rather than before habitual sleep time. Since melatonin data was available only for a portion of the 24 h cycle, it is not possible to fully ascertain whether the secretion of this hormone was truly delayed or only abnormally low (i.e. remaining below the DLMO threshold). In any case, dysregulation in either the timing or the level of melatonin release may be a key driver of circadian disorganisation in this subgroup of participants.

As opposed to the data-driven approach used here, most previous studies of circadian parameters reported global means or correlations. Consequently, it is possible that these studies indistinctively combined different circadian subgroups. The present findings sugget that people with delayed and disorganised circadian rhythms may constitute a clinically relevant subgroup in which psychiatric symptoms may relate more closely to circadian factors. Since the biological clock drives circadian rhythms in many other physiological functions (e.g. cortisol, heart rate), future studies should investigate circadian organisation across a wider range of phase makers in people with mood disorders.

In the present study, the ‘delayed’ cluster had more severe depressive symptoms than the ‘conventional timing’ cluster, and this was not driven by the HRSD sleep-related items. This is in line with a recent study showing that a subgroup of people with delayed sleep phase disorder who also had delayed DLMO had more severe depressive symptoms than those with more conventional DLMO timing^29^. Although none of the participants in the present study had a full blown bipolar illness, those who had a delayed circadian phase also appeared to have worse hypomanic symptoms. This link between circadian phase delays and both depressive and hypomanic symptoms further strengthen the hypothesis that circadian factors can interact closely with the symptomatology of mood disorders.

Several mechanisms potentially underlying the interactions between circadian and mood dysfunctions may be speculated. For example, circadian-driven alterations in the quality of wakefulness (e.g. extended morning inertia, low daytime energy levels, daytime sleepiness) and sleep (e.g. reduced nocturnal sleep propensity, sleep fragmentation) can lead to suboptimal affective functioning (for a review, see ref. ^30^). There have also been reports of unusual circadian period length^31^ and abnormal sensitivity to circadian synchronizers^32–35^ in people with mood disorders. Furthermore, circadian misalignment may also affect mood by altering monoamine signalling, hypothalamic pituitary adrenal (HPA) axis regulation, and neurogenesis, notably via dysregulations of the molecular clock^36,37^. These factors (alone or in combinations) may give rise to dynamic changes in circadian organisation. Hence, it would be relevant to direct further attention to the longitudinal patterns of circadian rhythms in young people with depressive disorders.

If circadian disruptions play a prominent role in the pathophysiology of depression for certain individuals, these individuals may be less responsive to standard antidepressant medications, and thus more prone to severe and persisting depressive symptoms. Close attention to circadian profiles may thus be helpful to optimise the management of mood disorders^38–40^, both for antidepressant treatments and preventive strategies in those at risk of developing a bipolar illness. The melatonin system is a promising therapeutic target, not only to readjust circadian phase, but also to restore circadian organisation. Exogenous melatonin, when ingested at the appropriate time, can phase shift endogenous melatonin, as well as CBT^41,42^. In addition, the melatonin system is highly responsive to environmental and behavioural manipulations (e.g. phase shifting sleep onset and offset times, light therapy, well-timed physical activity)^38^. Further work is required to determine whether chronotherapies may actively attenuate psychiatric symptoms severity or prevent transition towards more severe mental illness, and whether treatment response can be predicted by the initial circadian profile.

Limitations of the present study include: its cross-sectional nature precluding inferences about causality, relatively small sample size, and the fact that clinical scales data were not available for some participants. Some participants had psychiatric comorbidities and some were taking psychotropic medications, but these factors, which are common in people with depression, were not found to influence the circadian variables assessed in this study. Additionally, the control group was slightly but significantly older than the affective disorder group. However, there was no significant age difference across the identified clusters.

Overall, our findings unveiled a subgroup of young persons with unipolar depressive disorders presenting circadian phase delays and temporal disorganisation between circadian rhythms and the sleep–wake cycle. This subgroup was also characterised by worse depressive and hypomanic symptoms. Longitudinal studies are required to determine the relationship between circadian profiles and the course of illness of mood disorders. Pharmacological, environmental, or behavioural manipulations known to normalise circadian disorganisation may have therapeutic benefits for an identifiable subgroup of individuals with depressive disorders.

## Electronic supplementary material

Supplemental table 1
