# Supplementary material for: Circadian rhythms and psychiatric profiles in young adults with unipolar depressive disorders
Source: Transl Psychiatry. 2018 Oct 9;8:213. doi: 10.1038/s41398-018-0255-y (PMC6177460; doi:10.1038/s41398-018-0255-y)
Supplement: Supplementary file 1 — Supplemental table 1 [file 41398_2018_255_MOESM1_ESM.docx]

Supplemental table 1.

|  |  | Stratification Based on Comorbidities | | | | | | | | | | |  | Stratification Based on Psychotropic Medication Intake | | | | | | | | | | | | | |  |
| --- | --- | --- | --- | --- | --- | --- | --- | --- | --- | --- | --- | --- | --- | --- | --- | --- | --- | --- | --- | --- | --- | --- | --- | --- | --- | --- | --- | --- |
|  |  | No Comorbidities | | |  | Comorbidities | | |  |  | | |  | No Medicated | | | |  | | Medicated | | | |  | |  | | |
|  |  | n | Mean | SD |  | n | Mean | SD |  |  | t | p |  | n | Mean | SD |  | | n | | Mean | SD |  | |  | t | p | |
| DLMO |  | 12 | 24.2 | 3.2 |  | 23 | 23.9 | 2.7 |  |  | 0.28 | 0.780 |  | 16 | 24.8 | 2.5 |  | | 19 | | 23.4 | 3.0 |  | |  | 1.45 | 0.157 | |
| CBT_min_ |  | 12 | 30.1 | 2.7 |  | 23 | 28.9 | 2.1 |  |  | 1.42 | 0.164 |  | 16 | 29.2 | 2.3 |  | | 19 | | 29.4 | 2.5 |  | |  | -0.23 | 0.817 | |
| Sleep_ON_ |  | 12 | 24.5 | 1.1 |  | 23 | 24.6 | 1.7 |  |  | -0.10 | 0.919 |  | 16 | 25.1 | 1.2 |  | | 19 | | 24.2 | 1.7 |  | |  | 1.77 | 0.086 | |
| Sleep_OFF_ |  | 12 | 34.1 | 2.0 |  | 23 | 33.7 | 2.0 |  |  | 0.64 | 0.527 |  | 16 | 34.0 | 1.5 |  | | 19 | | 33.7 | 2.3 |  | |  | 0.38 | 0.704 | |
| Sleep_ON_-DLMO |  | 12 | 20.6 | 173.3 |  | 23 | 41.4 | 147.9 |  |  | -0.37 | 0.711 |  | 16 | 18.3 | 125.5 |  | | 19 | | 47.7 | 178.2 |  | |  | -0.55 | 0.583 | |
| CBT_min_-Sleep_mid_ |  | 12 | 44.6 | 125.5 |  | 23 | -15.2 | 104.1 |  |  | 1.50 | 0.142 |  | 16 | -18.9 | 131.8 |  | | 19 | | 25.6 | 94.9 |  | |  | -1.16 | 0.254 | |
| CBT_min_-DLMO |  | 12 | 352.2 | 126.1 |  | 23 | 298.9 | 176.5 |  |  | 0.93 | 0.360 |  | 16 | 265.9 | 178.7 |  | | 19 | | 360.3 | 134.6 |  | |  | -1.78 | 0.084 | |
|  |  |  |  |  |  |  |  |  |  |  |  |  |  |  |  |  |  | |  | |  |  |  | |  |  |  | |
| HDRS Total |  | 12 | 12.2 | 5.2 |  | 18 | 11.1 | 5.3 |  |  | 0.54 | 0.595 |  | 15 | 12.2 | 4.6 |  | | 15 | | 10.9 | 5.8 |  | |  | 0.70 | 0.493 | |
| Anxiety |  | 12 | 4.5 | 2.7 |  | 18 | 3.6 | 1.8 |  |  | 1.16 | 0.256 |  | 15 | 4.5 | 2.5 |  | | 15 | | 3.4 | 1.8 |  | |  | 1.35 | 0.188 | |
| Depression | | 12 | 5.4 | 2.9 |  | 18 | 5.4 | 3.5 |  |  | 0.02 | 0.982 |  | 15 | 5.5 | 2.5 |  | | 15 | | 5.3 | 3.8 |  | |  | 0.23 | 0.824 | |
| Insomnia | | 12 | 2.6 | 1.7 |  | 18 | 2.8 | 1.7 |  |  | -0.31 | 0.757 |  | 15 | 3.1 | 1.7 |  | | 15 | | 2.3 | 1.5 |  | |  | 1.47 | 0.152 | |
| Somatic |  | 12 | 2.3 | 1.1 |  | 18 | 2.2 | 1.5 |  |  | 0.17 | 0.869 |  | 15 | 2.2 | 1.4 |  | | 15 | | 2.2 | 1.3 |  | |  | 0.00 | 1.000 | |
| YMRS |  | 9 | 3.0 | 3.0 |  | 15 | 1.7 | 3.0 |  |  | 0.99 | 0.333 |  | 13 | 2.9 | 3.7 |  | | 11 | | 1.4 | 1.7 |  | |  | 1.34 | 0.196 | |

SD: Standard Deviation, DLMO: dim light melatonin onset, CBT_min_: core body temperature mimimum, Sleep_ON/OFF_: Sleep onset/offset, HDRS: Hamilton Depression Rating Scale total score (minus the sleep items) and subscales, YMRS: Young Mania Rating Scale.
